# Synthesis and characterization of Gd^3+^-loaded hyaluronic acid-polydopamine nanoparticles as a dual contrast agent for CT and MRI scans

**DOI:** 10.1038/s41598-023-31252-0

**Published:** 2023-03-18

**Authors:** Alireza Shariati, Tahereh Ebrahimi, Parva Babadinia, Fatemeh Sadat Shariati, Reza Ahangari Cohan

**Affiliations:** 1 Department of Materials Engineering, Tarbiat Modares University, Tehran, Iran; 2 Department of Nanobiotechnology, New Technologies Research Group, Pasteur Institute of Iran, Tehran, Iran; 3 Farzanegan High School, National Organization for Development of Exceptional Talents, Tehran, Iran; 4 Influenza Research Lab, Pasteur Institute of Iran, Tehran, Iran

**Keywords:** Health care, Medical research, Oncology, Nanoscience and technology

## Abstract

Magnetic resonance imaging and computed tomography (CT) suffer from low contrast sensitivity and potential toxicity of contrast agents. To overcome these limitations, we developed and tested a new class of dual contrast agents based on polydopamine nanoparticles (PDA-NPs) that are functionalized and targeted with hyaluronic acid (HA). These nanoparticles (NPs) are chelated with Gd^3+^ to provide suitable contrast. The targeted NPs were characterized through ultraviolet–visible spectroscopy (UV–vis), scanning electron microscopy (SEM), infrared Fourier transform (FTIR), and dynamic light scattering (DLS). The cytotoxicity was investigated on HEK293 cells using an MTT assay. The contrast property of synthesized Gd^3+^/PDA/HA was compared with Barium sulfate and Dotarem, as commercial contrast agents (CAs) for CT and MRI, respectively. The results illustrated that synthesized PDA-NPs have a spherical morphology and an average diameter of 72 nm. A distinct absorption peak around 280 nm in the UV–vis spectrum reported the self-polymerization of PDA-NPs. The HA coating on PDA-NPs was revealed through a shift in the FTIR peak of C=O from 1618 cm^−1^ to 1635 cm^−1^. The Gd^3+^ adsorption on PDA/HA-NPs was confirmed using an adsorption isotherm assay. The developed CA showed low in vitro toxicity (up to 158.98 µM), and created a similar contrast in MRI and CT when compared to the commercial agents. The r_1_ value for PDA/HA/Gd^3+^ (6.5 (mg/ml)^−1^ s^−1^) was more than Dotarem (5.6 (mg/ml)^−1^ s^−1^) and the results of the hemolysis test showed that at concentrations of 2, 4, 6, and 10 mg/ml, the hemolysis rate of red blood cells is very low. Additionally, the results demonstrated that PDA/HA/Gd^3+^ could better target the CD_44_^+^-expressing cancer cells than PDA/Gd^3+^. Thus, it can be concluded that lower doses of developed CA are needed to achieve similar contrast of Dotarem, and the developed CA has no safety concerns in terms of hemolysis. The stability of PDA/HA/Gd^3+^ has also been evaluated by ICP-OES, zeta potential, and DLS during 3 days, and the results suggested that Gd-HA NPs were stable.

## Introduction

MRI and CT scans are the most popular imaging techniques for diagnostic purposes. CT benefits from the advantages of fast screening speed, high spatial resolution, and low cost^1^. On the other hand, MRI is a non-invasiveness method with a radiation-free nature that can provide three-dimensional (3D) images of living organisms. Compared with other techniques, MRI has also advantages such as high flexibility in imaging, high resolution, excellent spatial resolution, better contrast for soft tissues, and non-use of ionizing rays^2^. Dotarem, Magnevist, Multihance, Optimark, and Gadovist are the most common commercial CAs in MRI^3^. According to the type of relaxation times, MRI contrast agents are classified as T1 or T2. The results of injecting gadolinium-based contrast agents (T1 CAs) into 100 million patients demonstrate a positive safety record as MRI CAs with high relaxivity at the visualization of tissues and organs. Meanwhile, the Gd^3+^-complex CAs with linear structures have disadvantages such as toxicity for healthy tissue (particularly in patients with kidney failure), low ability to chelate metal ions, high use of CAs to achieve sufficient contrast in imaging, and short blood retention time, which suggests the need to design and fabricate targeted CAs with better properties^4,5^. Therefore, the designing and manufacturing of a new gadolinium-based contrast agent with better properties than available contrast agents, such as dual contrast capability in CT and MRI, the ability to direct chelating metal ions, better biocompatibility, and most importantly targeting the tumor cell are essential. The dual CAs are used in approximately half of all examinations. Combining the data obtained from imaging modalities can significantly improve the diagnosis of diseases. Moreover, these dual CAs provide volumetric data at orientations and various views (in both 2D and 3D) for better and rapid diagnosis. Clinical studies show that the diagnosis of diseases such as acute stroke, tumor detection, and vulnerable plaques can be improved by sequential scanning in both MRI and CT imaging. These dual CAs can increase the flexibility of imaging systems, so the development and construction of a single agent with dual contrast characteristics in both methods for the diagnosis and treatment of diseases are of great importance^6^.

In recent decades, the integration of NPs has dramatically affected medical imaging such as CT and MRI^7,8^. The use of NPs as a chelator has several advantages over small molecules including cell tracking capacity, targeted imaging, carrying higher amounts of CAs, and a long half-time in the bloodstream. High payloads of gadolinium ions in these NPs provide more sensitivity to detect diseased tissue within the human body. In previous studies, different NPs were employed to synthesize the CAs. Nonetheless, the use of these NPs is associated with many limitations. For example, although the biocompatibility of dendrimers can be improved with polyethylene glycol coupling, it leads to a reduction in the relaxation rate^9^. Carbon-based nanocarriers have high proton relaxivity, but their application has safety considerations^10,11^. The release of chelated gadolinium ions from fluorescent nanotubes in tissues causes severe health problems^11^. Toxicity and biocompatibility concerns about AuNPs are also evident and the use of these NPs for the synthesis of CAs is not cost-effective^12–14^. Silica-based NPs offer good biocompatibility, but like other CAs, they require a chelator for the loading of paramagnetic ions^9^. Liposomes are widely used as CAs, however, their apparent instability within the body is their main weakness. Moreover, the slow flux of water across the membrane bilayer impairs the water exchange rate with encapsulated Gadolinium ions which leads to a significant reduction in relaxivity^9,13^. Cholesterol, which is generally required to increase the stability of liposomes, further reduces the relaxivity of encapsulated Gd^3+^-PEGylated liposomes^15^. At the same time, the cost of producing these nanocarriers is relatively high, and it is difficult to prepare them on a large scale^16^.

Currently, one of the widest nanomaterials used in medicine is polymeric NPs which are applied as carriers or targeting agents for therapeutic or diagnostic purposes^17^. Among them, dopamine with the self-polymerization property has unique advantages including high biocompatibility, easy and economical synthesis, as well as the ability to load multiple molecules for various applications^18^. PDA-NPs can adhere to all surfaces, even underwater, owing to the presence of abundant catechol, indole, and pyrrole. This property, together with its broad reactivity to nucleophiles and electrophiles, has made them interesting for various applications in biology and biomedicine^7,8,18–21^.

In the design of CAs, targeting is necessary to produce detectable changes in the signal intensity of the target tissue via altering the relaxation property^22^. Different targeting moieties including natural or synthetic polymers could be utilized for this purpose^22–26^. HA, a natural ligand for the CD44 receptor which is overexpressed in cancer cells is popularly utilized as a targeting moiety^27,28^. In addition to its inherent targeting ability, this polymer also has several desirable properties including non-toxicity to living organisms, biodegradability, low immunogenicity, and non-inflammatory property in medical programs^29–31^.

**Figure 1:**
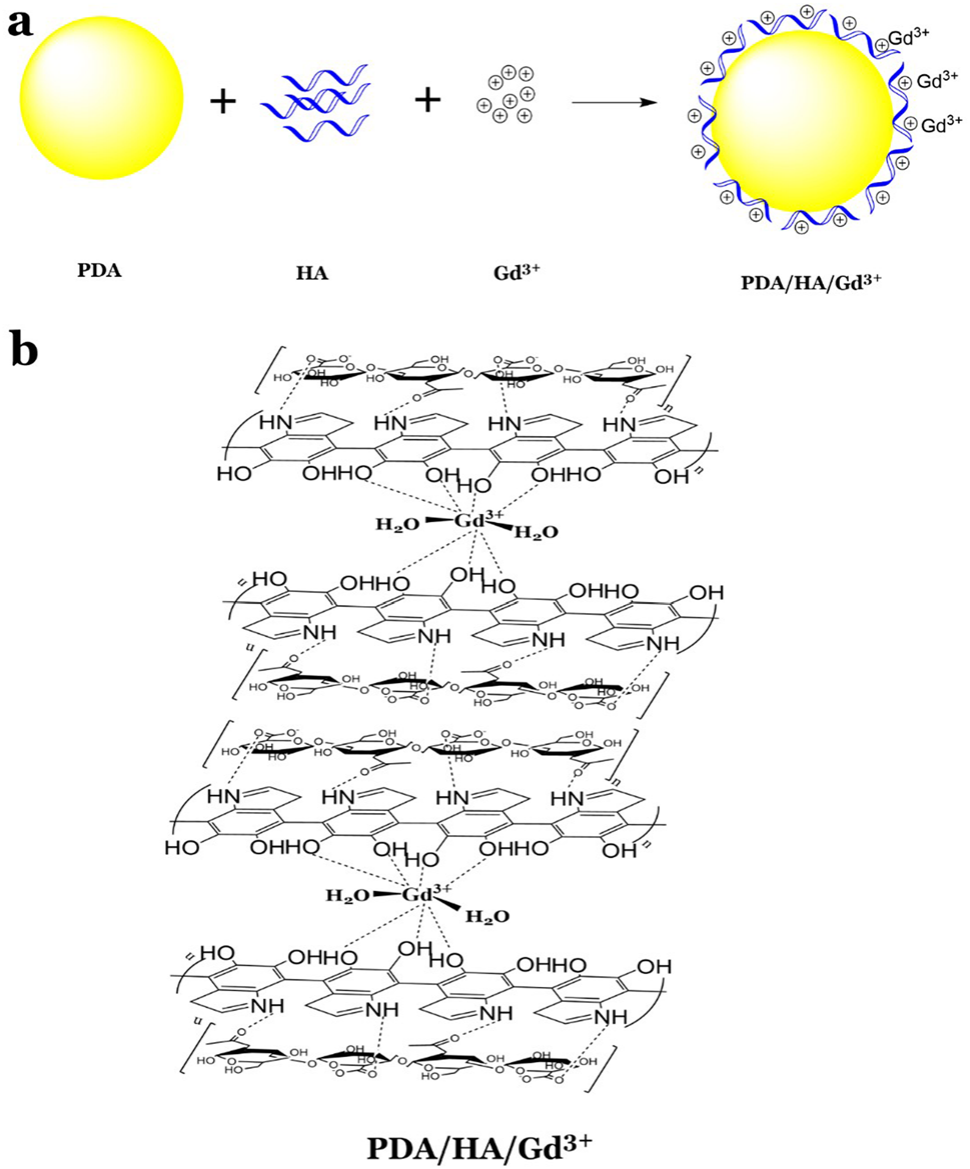
PDA/HA/GD3 + image. (**a**) The synthesis procedure of Gd^3+^-dual contrast agent. (**b**) The chemical structure of prepared Gd^3+^-loaded PDA/HA-NPs.

Accordingly, this study aimed to synthesize and characterize a dual contrast agent based on PDA-NPs targeted with HA and chelated with Gd^3+^ for MRI and CT imaging (Fig. 1). Gd^3+^/PDA/HA-NPs can potentially serve as an efficient contrast agent for improved contrast of MRI and CT as two different imaging techniques with particular importance.

## Results

### UV–visible analysis

**Figure 2:**
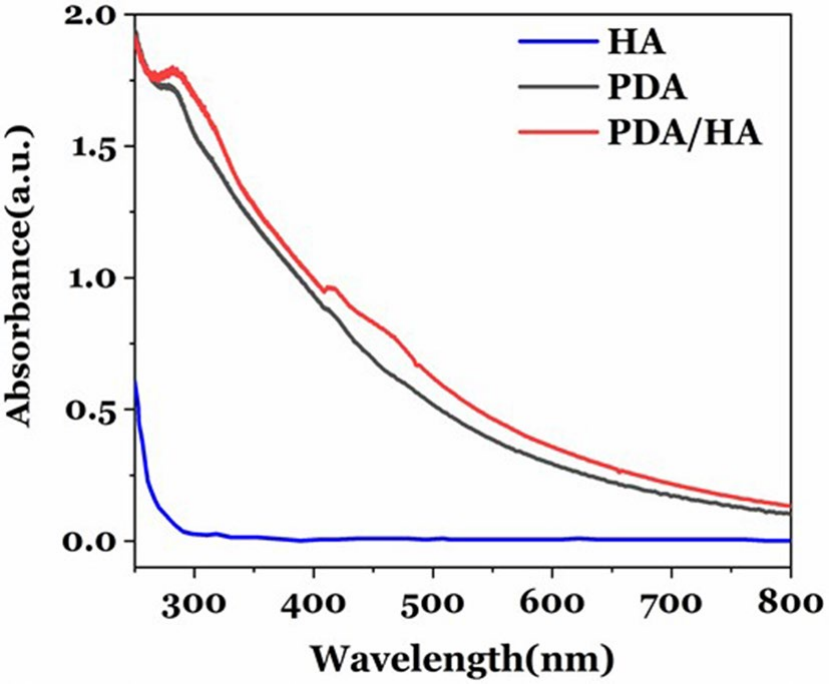
UV–Visible analysis. UV–Visible spectra of synthesized PDA/HA-NPs, PDA-NPs, and HA.

Figure 2 shows the UV–visible spectra of PDA/HA-NPs, PDA-NPs, and HA. A peak at 280 nm is observed for the synthesized PDA/HA-NPs and PDA-NPs, however, it is absent in the HA spectrum. This confirms the formation of PDA-NPs in the presence of HA.

### SEM and DLS analysis

**Figure 3:**
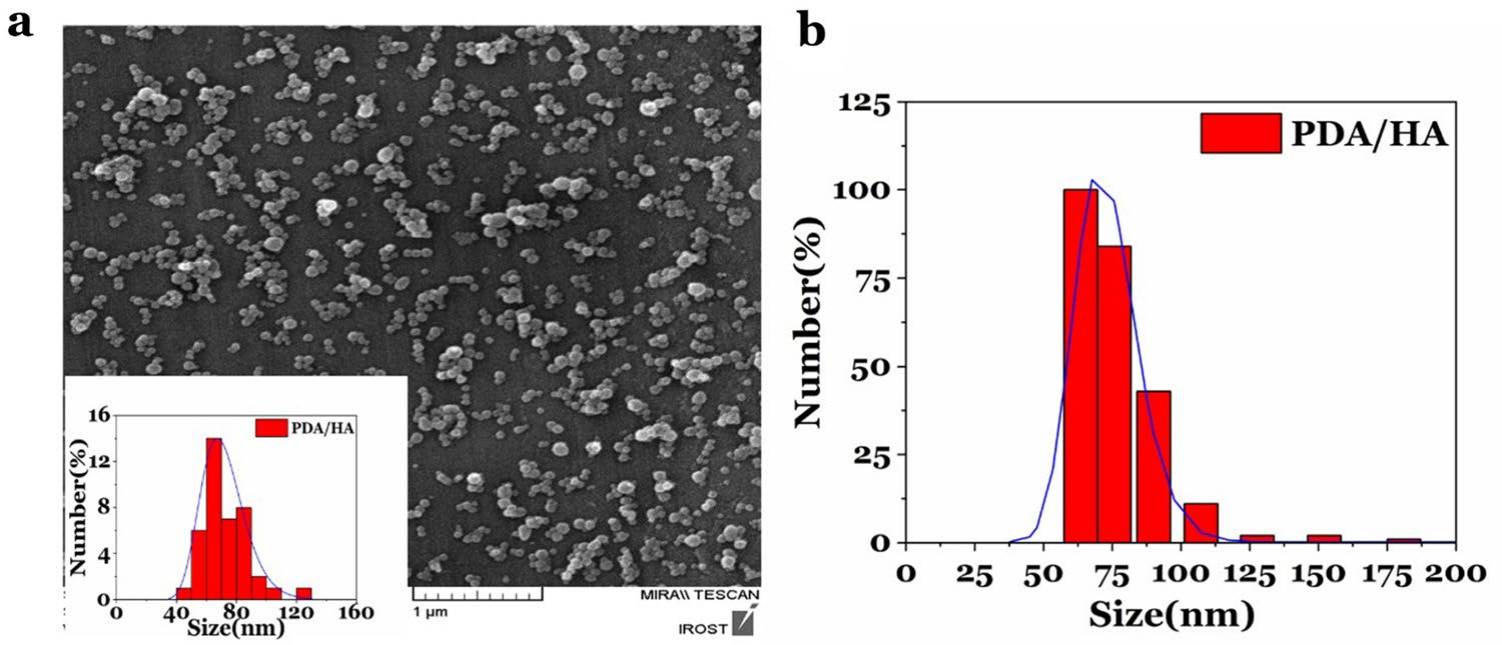
SEM analysis. (**a**) The SEM image and the average size histogram of PDA/HA-NPs. (**b**) The hydrodynamic size distribution of PDA/HA-NPs using the DLS technique.

SEM analysis revealed the PDA/HA-NPs had a spherical morphology (Fig. 3a). The average diameter of 40 particles was randomly measured on SEM images via Image J software. The results showed that the average size of 40 particles was ~ 70 nm (Fig. 3a). Additionally, the average hydrodynamic size distribution of PDA/HA-NPs was measured with DLS which obtained ~ 72 nm (Fig. 3b).

### FTIR analysis

**Figure 4:**
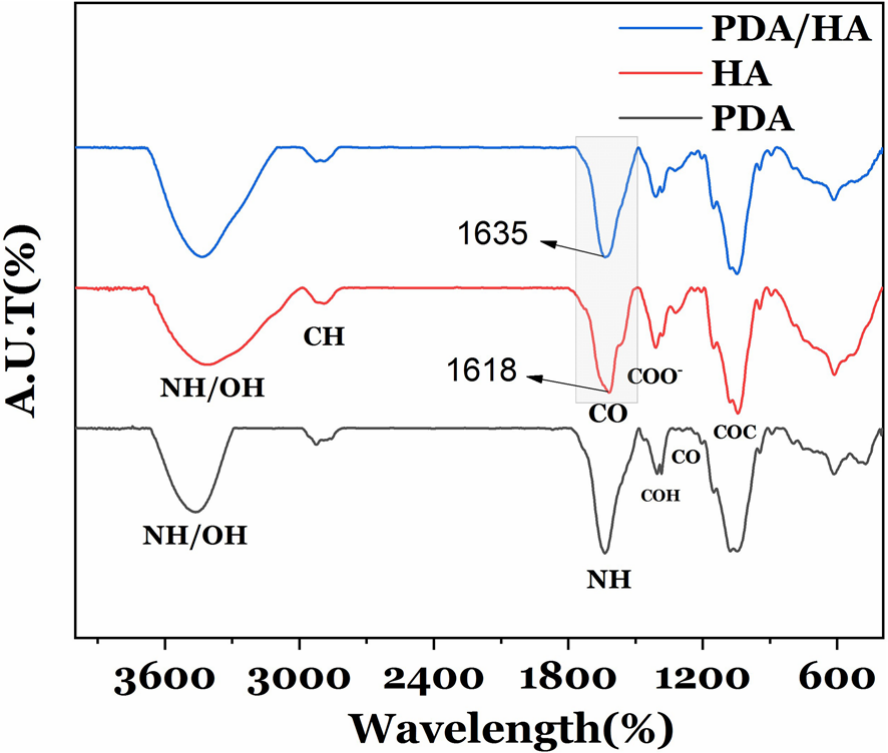
FTIR analysis. FTIR of synthesized PDA/HA-NPs, PDA-NPs, and HA.

HA loading on PDA-NPs was confirmed via FTIR spectrum analysis (Fig. 4). The tensile vibrations of OH and NH bonds in 3716–2993 cm^−1^ are related to hydroxyl amide and secondary carboxylic acid bonds. An absorption peak at 2894 cm^−1^ is related to the alkane CH (stretching) bond. The absorption peaks at 1618 cm^−1^ and 1044 cm^−1^ can be attributed to C=O stretching of carbonyl groups and C–O–C symmetric stretching, respectively. The HA aromatic groups can be observed at 615 cm^−1^ in the spectrum. For PDA-NPs, the peak at 1288 cm^-1^ is related to C–O tensile vibration. Flexural and tensile vibrations of OH were observed at 1371 cm^−1^ and 1341 cm^−1^, respectively. An observed peak at 1637 cm^−1^ is related to the N–H vibration of the amine group. A wide peak, which appeared between 3200 cm^−1^ and 3500 cm^−1^, also shows the OH and N–H bonds in the catechol groups. As Zhou et al. and Kim et al*.* reported, there is a strong electrostatic interaction and hydrogen bonding between PDA and HA molecules. This hydrogen bond is between the O atom of the amide and carboxyl groups of HA and the H atom of the PDA amine group (NH–CO). In PDA/HA spectrum, a shifting peak of C=O from 1618 cm^−1^ to 1635 cm^−1^ confirms the existence of a hydrogen bond between the C=O of HA polymer and the N–H amine group of PDA.

### Adsorption study

**Figure 5:**
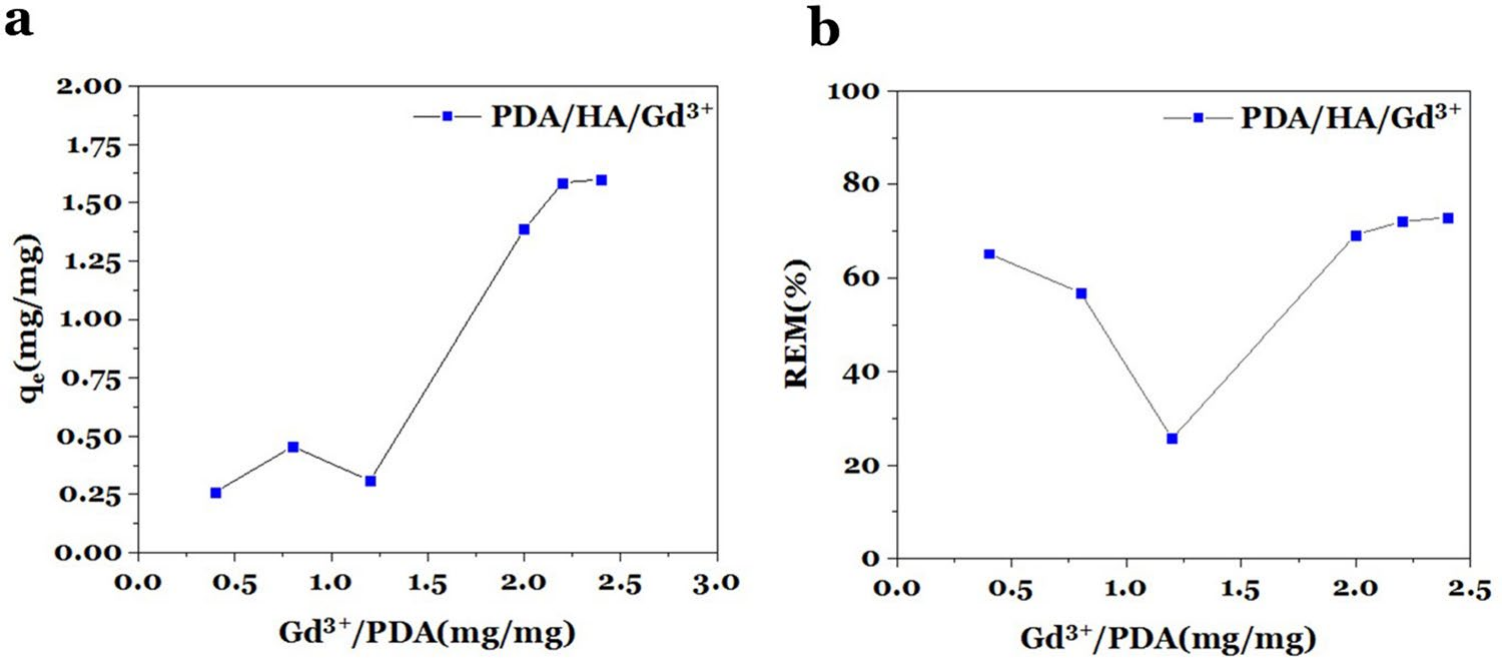
Adsorption study. Ion adsorption (q_e_) and adsorption efficiency (REM %) measurements. (**a**) Adsorption graph of Gd^3+^ ions on PDA/HA-NPs, and (**b**) Gadolinium ion adsorption efficiency of PDA/HA-NPs.

The adsorption (q_e_) of the gadolinium ions on PDA/HA-NPs at different ion:PDA-NPs weight ratios (0.4:1, 0.8:1, 1.2:1, 2.0:1, 2.2:1, and 2.4:1) were measured using ICP-OES (Fig. 5a). In the adsorption plot, with the increasing weight ratio of Gd^3+^ ion to PDA/HA, the slope was also increased, but from the weight ratio of 2.2 onwards, the diagram showed a steady slope. This means that the weight ratio of 2.2:1 is economical in terms of material and can be used for MRI and CT. Moreover, the adsorption efficiency (REM%), which means the Gd^3+^ ions removed relative to the initial value, was calculated based on the result of ICP-OES by repeating the experiment in triplicate (Fig. 5b).

**Figure 6:**
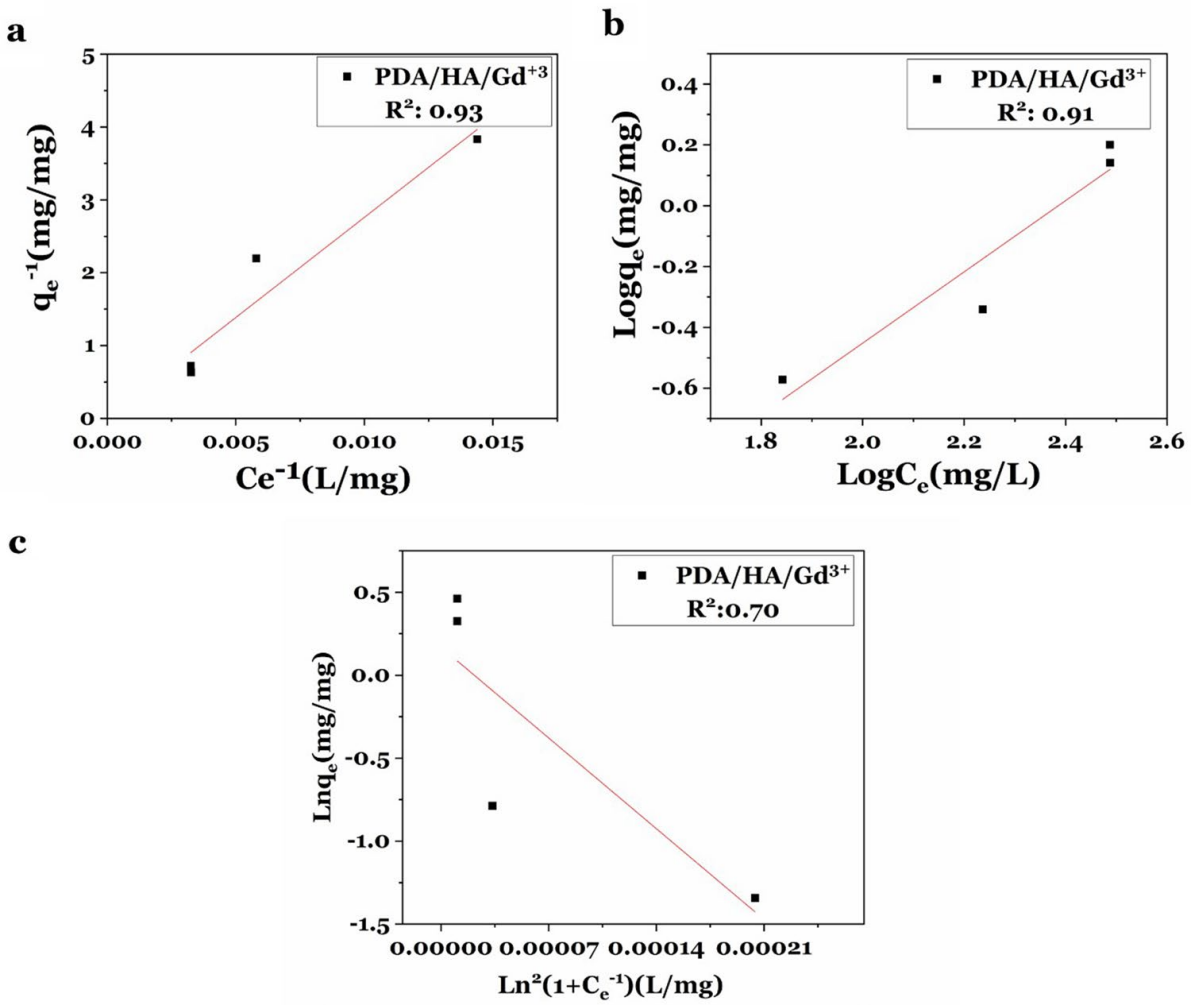
Adsorption isotherm study. (**a**) Langmuir adsorption plot, (**b**) Freundlich plot, and (**c**) Dubinin-Radushkevich plot.

Subsequently, Langmuir, Freundlich, and Dubinin-Radushkevich (D-R) isotherm equations were used to describe the equilibrium between adsorbed Gd^3+^ ions and those remaining in the aqueous solution. q_e_^-1^ and C_e_^-1^ (defined in Table 1) were calculated for all weight ratios. According to the Langmuir equation and Langmuir diagram, the value of q_max_^-1^ equals 0.01075 and k_L_ (defined in Table 1) equals 3.95 × 10^–5^ (Fig. 6a). R_L_ or separation factor for ratios of 0.4:1, 0.8:1, 1.2:1, 2.0:1, and 2.2:1 equal 1.02, 1.03, 1.04, 1.05, and 1.06, respectively (R_L_ = 1.02–1.06). Log q_e_ and log C_e_ (defined in Table 1) were calculated for all weight ratios according to the Freundlich equation and plotted (Fig. 6b). According to Freundlich, the value of n_f_ (defined in Table 1) equals 0.85. Therefore, Langmuir and Freundlich indicated that the adsorption of Gd^3+^ ions on PDA/HA-NPs is an unfavorable process. Moreover, Ln q_e_ and Ln^2^ (1 + C_e_^-1^) (defined in Table 1) were calculated for all weight ratios. According to the Dubinin-Radushkevich equation, ln q_e_ was plotted in terms of Ln^2^ (1 + C_e_^−1^) (Fig. 6c). Based on Dubinin-Radushkevich plot, the amount of adsorption energy (E) was calculated at 35 kJ mol^−1^, which was higher than 8 kJ mol^−1^. This indicates the chemisorption nature of the adsorption process.

**Table 1:** The Langmuir, Freundlich, and Dubinin-Radushkevich isotherm equations.

Isotherm	Equation	Definition	References
The Langmuir isotherm	$\frac{1}{{q_{e} }} = \frac{1}{{q_{\max } K_{L} }}\frac{1}{{C_{e} }} + \frac{1}{{q_{\max } }}$	**q**_**max**_** (mg g**^**−1**^**):** The maximum adsorbent capacity **K**_**L**_** (L mg**^**−1**^**):** The constant of Langmuir isotherm equation^44^	^45^
The dimensionless separation factor (R_L_)	$R_{L} = \frac{1}{{1 + K_{L} C_{0} }}$	If 0 < **R**_**L**_ < 1, favorable adsorption, R_L_ > 1, unfavorable adsorption, **R**_**L**_ = 1, linear adsorption, and **R**_**L**_ = 0, irreversible adsorption	
Freundlich isotherm	${q}_{e}={K}_{F}\times {{C}_{e}}^{\frac{1}{{n}_{F}}}$	K_F_ (mg g^**−**1^) and n_F_ are described as dimensionless **n**_**F**_** value:** The determination of the affinity when the adsorption process is unfavorable (n_F_ < 1), and when the adsorption is favorable (n_F_ > 1)^44,46^	^47^
Dubinin-Radushkevich isotherm	$$\begin{gathered} \ln q_{e} = \ln q_{s} - B\varepsilon^{2} \hfill \\ \varepsilon = RT\ln \left( {1 + \frac{1}{{C_{e} }}} \right) \hfill \\ \end{gathered}$$	**q**_**s**_** (mg g**^**−1**^**)**_**:**_ The theoretical maximum adsorption capacity of Dubinin-Radushkevich (D-R) ${\varvec{B}}$ **(mol**^**2**^** kJ**^**−2**^**):** A D-R model constant that is related to the mean free energy of adsorption per mole of the adsorbate **ɛ (Kj mol**^**−1**^**):** Polanyi potential that is defined as an equation^44,46,48^	^44,46,48^
The average free adsorption energy	$E = \left( { - 2B} \right) ^{ - 0.5}$	**R (kJ.K**^**−1**^** mol**^**−1**^**):** The constant of gases **T:** The temperature (Kelvin) **Adsorption energy (kJ mol**^**−1**^**):** Provides information about physical and chemical properties Values of E between 8 and 16 indicate the adsorption process of chemical ion exchange Values of E < 8 are the adsorption process of the physisorption type Values of E > 8 are the adsorption process of the chemisorption type

### MRI and CT scans

**Figure 7:**
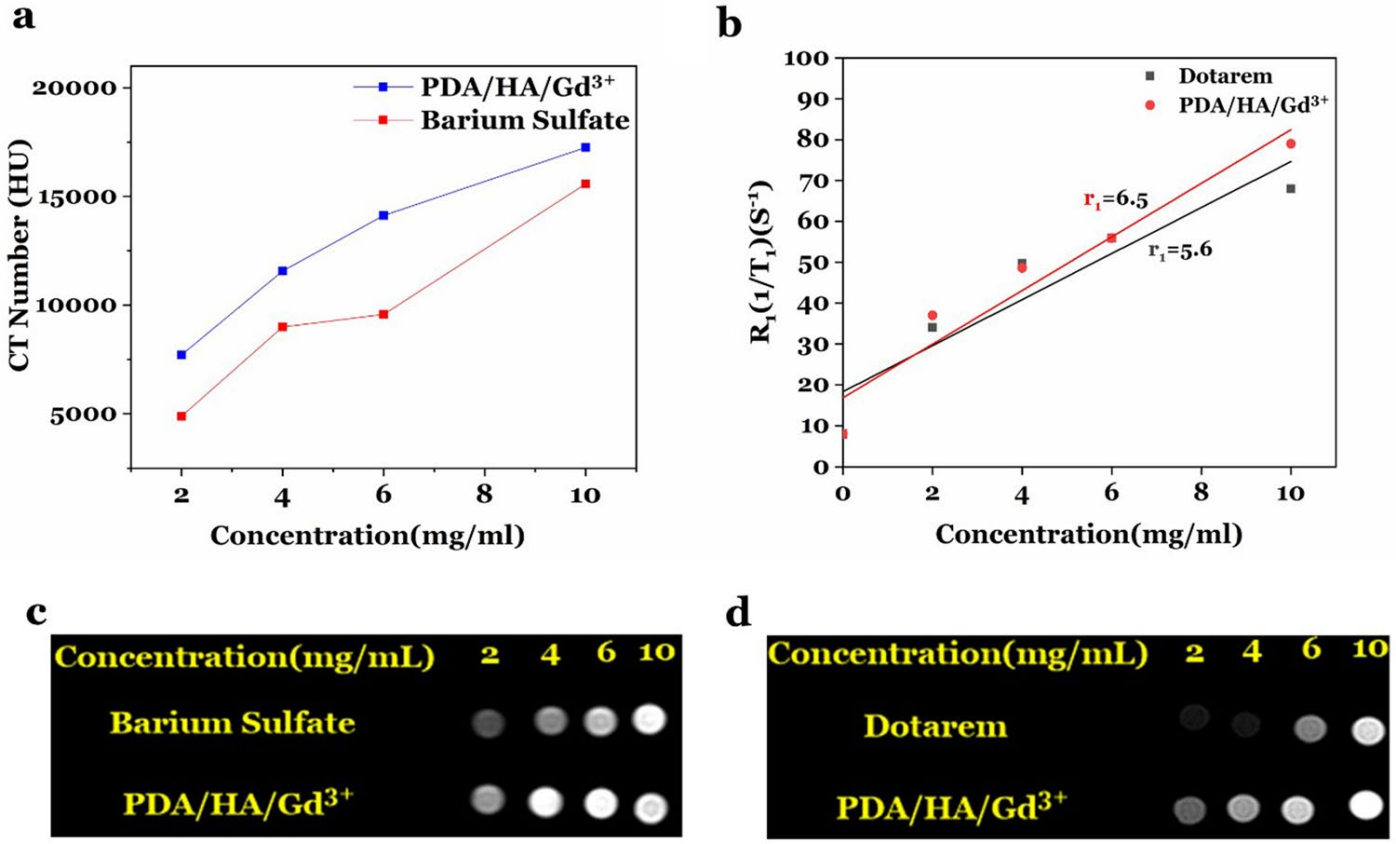
MRI, CT, and T1 weighted MRI plots. (**a** and** c**) The CT number plot and the signal rate of PDA/HA/Gd^3+^ NPs at different concentrations (2, 4, 6, and 10 mg/ml) were compared with barium sulfate (as a commercial CT contrast-positive control), respectively. (**b** and** d**) T1 weighted MRI plot and the signal rate of PDA/HA/Gd^3+^ NPs at different concentrations (2, 4, 6, and 10 mg/ml) in comparison to Dotarem (as a commercial MRI contrast-positive control).

MRI and CT scans were performed using PDA/HA/2.2 Gd^3+^ at different concentrations of gadolinium ions (0, 2, 4, 6, and 10 mg/ml). This concentration range was selected based on the hemolysis results, in which Gd^3+^ has weak hemolysis. Finally, the results were compared with the commercial contrast agents (Fig. 7). The results showed the signals acquired from the synthesized contrast agent in MRI are higher than its commercial agent at an equal concentration (Dotarem as positive control). Also, in the CT scan, the CT number of the synthesized CA is approximately 1.5 times more than BaSO_4_ (the commercial contrast agent: positive control) in all concentrations. The T1 weighted MRI Plot (Fig. 7b) showed that r_1_ value for PDA/HA/Gd^3+^ (6.6 (mg/ml)^−1^ s^−1^) was more than Dotarem (r_1_:5.6 (mg/ml)^−1^ s^−1^). Thus, it can be concluded that lower doses of developed CA are needed to achieve similar contrast to Dotarem.

### MTT assay

**Figure 8:**
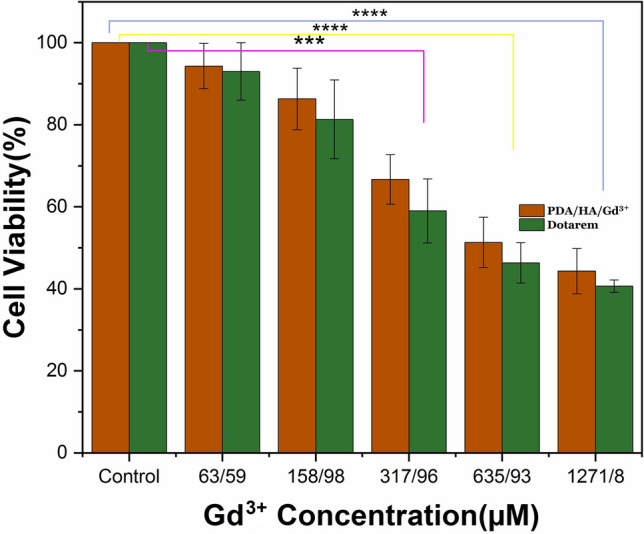
The cytotoxicity of Gd^3+^ ions adsorbed on PDA/HA/NPs on HEK293 cells using MTT assay. Data are demonstrated as mean ± SD (n = 3). The significant difference levels are shown with asterisks compared to the negative control (**** and *** means *p*-value less than 0.0001 and 0.001, respectively).

The cytotoxicity of PDA/HA/Gd^3+^ NPs was evaluated on HEK 293 cells using an MTT assay (Fig. 8). The results showed that the cells kept surviving even after 72 h when the gadolinium concentration adsorbed on PDA/HA/Gd^3+^ NPs is below 158.98 µM. In this study, the treatment time of the CAs with the cells was 72 h, which can reduce cell viability, especially at high concentrations of CAs. This issue is in agreement with previous studies that cell viability decreases with increasing treatment time^6^. In addition, in the previous studies conducted on the toxicity of gadolinium complexes, the results showed that the toxicity of contrast agents is in the range of micrograms, and the synthesized CA in our study has less toxicity or is similar to the other available CAs^11,32^.

### Hemolysis test

**Figure 9:**
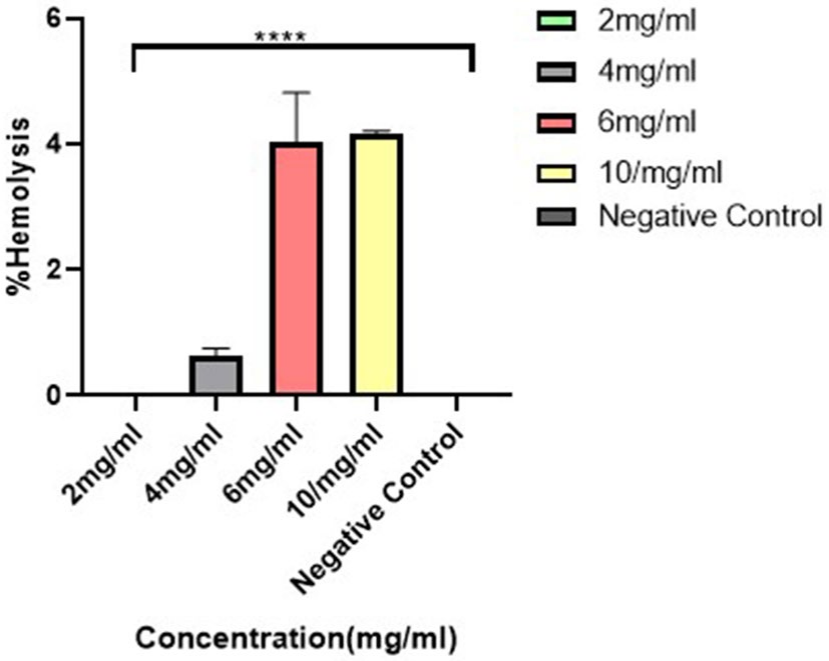
Hemolysis plot. The plot shows the percentage of hemolysis after the treatment of samples with different concentrations of PDA/HA/Gd^3+^ and PBS (negative control). Data are demonstrated as mean ± SD (n = 3). The significant difference levels are shown with asterisks compared to the negative control (**** means a *p*-value less than 0.0001).

The supernatants for PDA/HA/Gd^3+^ and the PBS remained clear, while the distilled water sample (positive control) turned red color due to the breaking of red blood cells (hemolysis phenomenon). Also, the OD_550nm_ difference between PDA/HA/Gd^3+^ and negative control was significant at all concentrations. A hemolysis rate of 2% or less is considered excellent for CAs, while a rate of less than 5% is considered low hemolysis^33^. Thus, the developed CA did not hemolyze red blood cells at concentrations of 2, 4, 6, and 10 mg/ml, and is safe (Fig. 9).

### Targeting study

**Figure 10:**
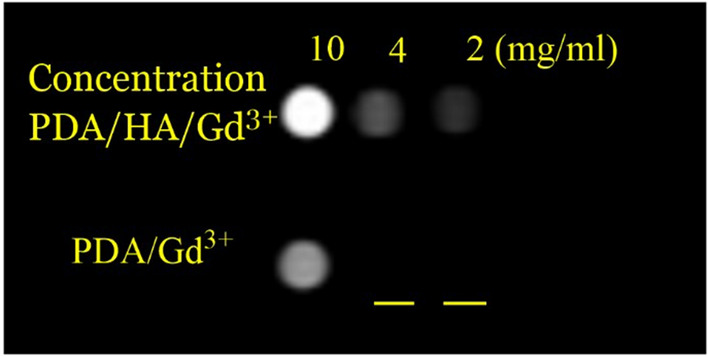
In vitro targeting MRI test. In vitro T1 MRI images of MDA-MB-231 cells treated with the PDA/HA/Gd^3+^ and PDA/Gd^3+^ CAs.

The targeting study was conducted by measuring the cellular uptake of HA-decorated CA following incubation with the breast cancer cells for 4 h at 37 °C. The fluorescence intensity of PDA/HA/Gd^3+^ NPs was higher in all concentrations than in the control sample. An increase in the signal intensity can be attributed to the interaction of hyaluronic acid with the CD_44_ receptor on the surface of cancer cells, which means more cell internalization for PDA/HA/Gd^3+^ than PDA/Gd^3+^ (Fig. 10).

### The stability study of PDA/HA/Gd^3+^

**Figure 11:**
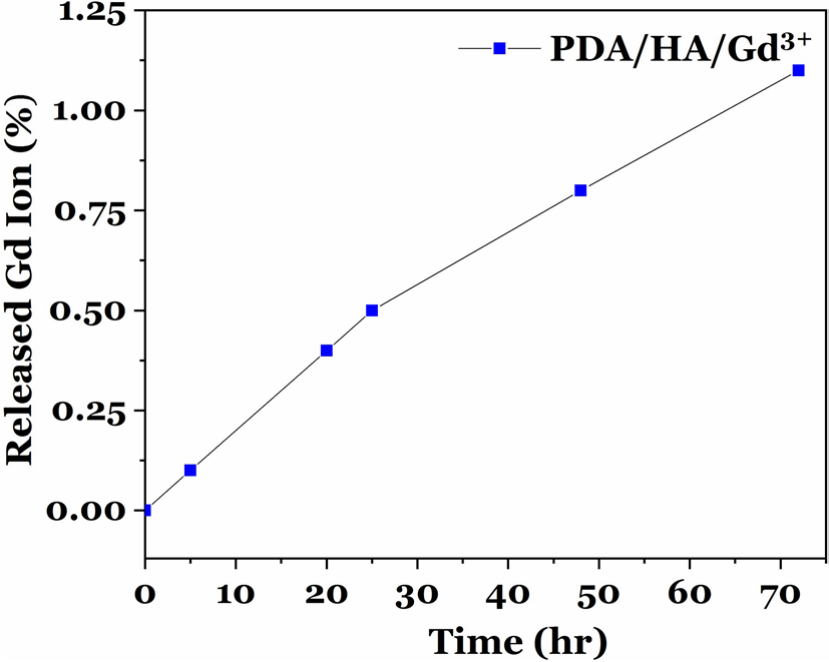
The percentage of gadolinium ions released from PDA/HA/Gd^3+^ NPs during 72 h.

The stability of PDA/HA/Gd^3+^ has also been evaluated by measuring free Gd^3+^ ions in the supernatant after 72 h filtering with a dialysis bag (cutoff: 12,000–14,000 Da) against deionized water (DI) for 3 days. The result of ICP-OES (Fig. 11) showed ~ 1% of free ions in the supernatant after 72 h.

**Figure 12:**
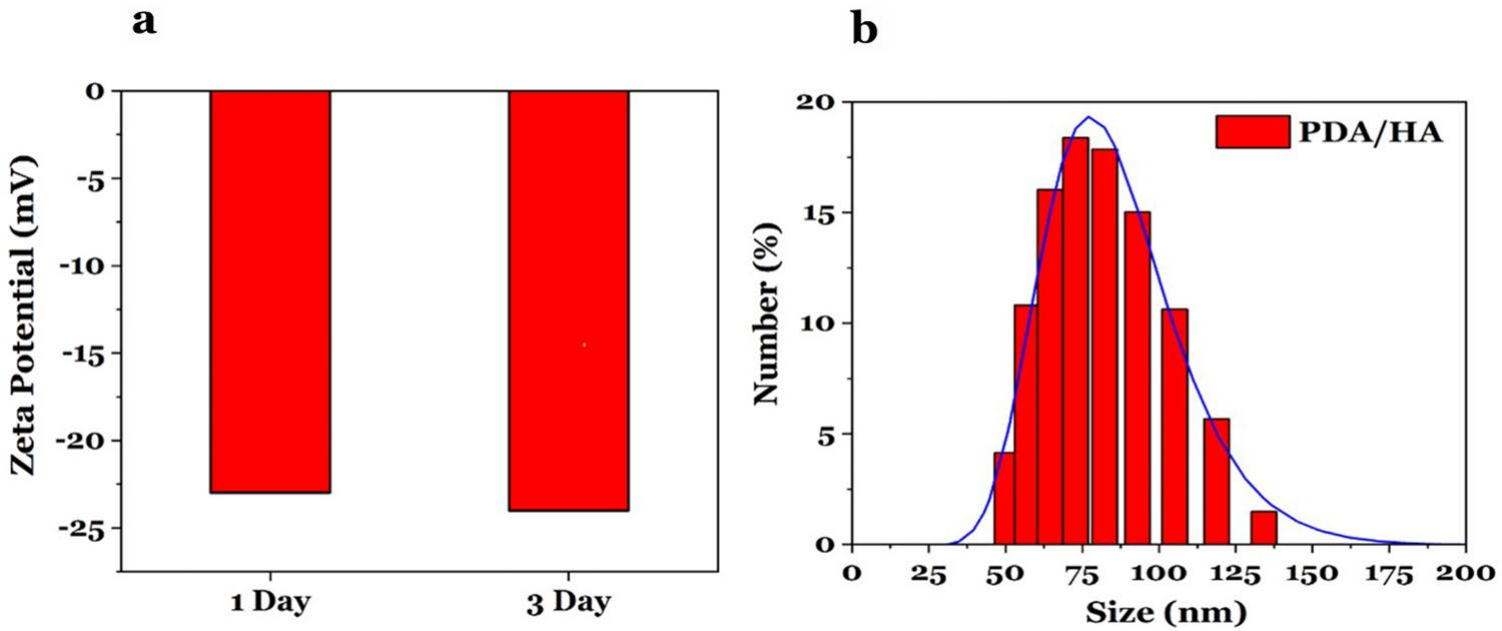
Stability test of PDA/HA/Gd^3+^ NPs in human serum at 37 °C for 3 days. (**a**) The hydrodynamic diameter of the NPs at days 1 and 3. (**b**) The zeta potential of the NPs at days 1 and 3.

Moreover, to evaluate the stability of PDA/HA/Gd^3+^, the changes in the size and surface charge of NPs were measured through hydrophilic size measurements (DLS) and the zeta potential at days 1 and 3 (Fig. 12). The results showed that the size of PDA/HA/Gd^3+^ NPs has slightly increased from 72 nm to ~ 78 nm after three days which is not significant. The zeta potential of the PDA/HA/Gd^3+^ changed from − 23 mV to − 24 mV after three days, and the results showed no negligible changes during incubation. Thus, the results suggested that PDA/HA/Gd^3+^ NPs were stable.

## Discussion

In this paper, we have synthesized PDA/HA/Gd^3+^ dual CA that showed a good MR and CT contrast when compared to Dotarem and Barium sulfate as commercial CAs. The PDA-NPs had low cytotoxicity, and relatively high solubility and stability in water. Moreover, the coating of these NPs with HA can target the CAs to the CD_44_-overexpressing cells. SEM analysis determined the morphology of synthesized NPs as spherical with a diameter of ~ 70 nm. The hydrodynamic size of PDA/HA-NPs also showed a size distribution of 72 nm. Thus, the minor difference in the reported mean with the two methods could be due to the non-recognizable boundaries of NPs in the SEM image. Moreover, dynamic light scattering (DLS) is a technique that measures the hydrodynamic diameter of the particle including the solvation layers^34–36^. Studies by Xue et al*.* and Kim et al*.* indicated that there is a strong electrostatic interaction and hydrogen bonding between HA and PDA molecules. They reported the hydrogen bond constituted between the oxygen atom of amide/carboxyl groups of HA and the hydrogen atom of amine groups on PDA-NPs (N–H–O=C). Similarly, in this study, the coating of the nanoparticle surface with HA was confirmed with FTIR analysis through a shift in the peak of C=O from 1618 cm^−1^ to 1635 cm^−1^ which confirms the existence of a hydrogen bond between the C=O of HA polymer and the N–H amine groups on PDA.

The similar UV–vis spectra of PDA-NPs and PDA/HA-NPs indicated that PDA coating with HA did not affect the optical properties of NPs. The PDA-NPs and PDA/HA-NPs have an obvious optical absorption peak at 280 nm. This absorption peak confirmed the formation of PDA-NPs in the presence of HA when compared with the HA spectrum.

The result revealed the PDA/HA/Gd^3+^ has higher signal intensity and CT number than commercial contrast agents (Dotarem for MRI and Barium sulfate for CT). In detail, the CT number of PDA/HA/Gd^3+^ contrast agent was almost close to the Barium sulfate at the same concentration, and as expected, the X-ray attenuation enhanced with the increase in the Gd^3+^ concentration. In other words, PDA/HA/Gd^3+^ NPs could produce the same or better x-ray attenuation than the Barium sulfate at the same concentration. In T1 weighted MRI plot, the result showed that r_1_ value for PDA/HA/Gd^3+^ (6.5 mg/ml^−1^ s^−1^) was higher than Dotarem (r_1_:5.6 mg/ml^−1^ s^−1^). Therefore, it can be concluded that lower doses of developed CA are needed to achieve similar contrast to Dotarem. Although the mechanism for the high relaxivity of PDA/HA/Gd^3+^ is still not well understood, it is often described by the Solomon, Bloembergen, and Morgan (SBM) theory. According to this theory, the high relaxivity of the new dual contrast can be explained by the mean residence lifetime of the coordinate waters (TM), the rotational correlation time (TR), and most importantly the number of water molecules directly coordinated to the paramagnetic center (q) in the inner sphere (than those at a greater distance in the second and outer spheres). Gd(III) is a paramagnetic element that bounds with nearby water molecules with a fluctuating magnetic field, decreases their T1 and T2 relaxation times, and increases the bright contrast in MR imaging. Commercial Gd(III) contrast agents with a relaxivity of approximately four usually have one coordinate water molecule (q = 1). As inferred from previous studies, in PDA/Gd3^+^ NPs due to having an enhanced surface area accessible for water molecules, these molecules can directly contact the paramagnetic center, and this interaction leads to the transmission of relaxivity. In addition, in these CAs according to a second sphere-like mechanism, a large number of water molecules interacted with the functional groups in the nanostructure surface that increase the probability of exchanging water protons. On the other hand, in a previous study, it was reported PDA plays a key role in water proton relaxation^11,37–40^. The signal intensity in MRI is related to the relaxation times (T1, spin–lattice relaxation, and T2 spin–spin relaxation) of in vivo water protons. The complexes of gadolinium ions, characterized by seven unpaired electrons in the valence 4f. orbital, are associated with a high spin quantum number (*S* = 7/2), high magnetic moment, symmetric orbital ground states, large longitudinal electronic relaxation times (∼10^−8^ s), and faster water exchange kinetics than Mn^2+^ and Fe^3+^, which have five unpaired electrons in the d subshell. The results obtained by a previous study showed that Gd^3+^ or Gd^3+^-complex when incorporated with nanomaterials improves their longitudinal relaxivity through an increase in the rotational correlation time, and has gradual and elongated signals due to the slow release and cellular uptake, which subsequently enhances the permeation and retention (EPR) effect. For example in a study by Le Duc et al.polysiloxane-encapsulated Gd_2_O_3_ NPs showed ongoing MRI signal 24 h after injection of CAs into the tumor due to the slow release^41,42^.

In a study by Chen et al. polydopamine-coated gold NPs were used as a contrast agent for MRI and x-ray tomography. By coating a layer of polydopamine on the surface of gold NPs, the light conversion efficiency was significantly improved, which was confirmed through a CT scan. These gold NPs coated with polydopamine showed optimal visible properties, however, the use of gold NPs as a contrast agent entails safety considerations and is not cost-effective. Also, our result is in accordance with the previous study by Miao et al. in which PDA-NPs chelated with Mn^2+^ ions improved the MRI signal^19^. Recently, several HA-conjugated Gd^3+^ chelates have been synthesized to enhance the MRI contrast. For example, Guo et al*.* prepared dendronized-HA-tetraazacyclododecane-1, 4, 7, 10-tetraacetic acid-Gd^3+^ that exhibited high sensitivity for tumor diagnosis^43^. In a study by moon et al*.* Gd^3+^ ions were integrated with DTPA (diethylenediamine pentaacetate) and hyaluronic acid (HA) for synthesizing DTPA/HA/Gd^3+^ NPs to improve the quality of MRI contrast for hepatic cells^43^. We also showed that PDA/HA/Gd^3+^ CA can directly target cancer cells that overexpressed the CD_44_ receptors.

## Conclusion

In the current study, a novel nano-based contrast agent was successfully synthesized, and the cytotoxicity, stability, as well as efficiency of use for MRI and CT scans, were investigated. MRI and CT scan elucidated that the synthesized dual contrast agent is effectively equal to or more than the commercially available contrast agents. Moreover, the developed contrast agent did not show in vitro cytotoxicity at a used dosage and was stable. Therefore, it can be considered an efficient multifunctional contrast agent platform for targeting CD_44_^+^-overexpressing cancer cells.

## Experimental sections

### Materials and characterizations

Dopamine hydrochloride (DH), Tris–HCl, and all other chemicals were purchased from Sigma-Aldrich, USA. Sodium Hyaluronate with a molecular weight of 8000–15,000 Daltons was obtained from Making Cosmetics, USA. The morphology and size distribution of prepared NPs were monitored with scanning electron microscopy (SEM) (MIRA3, Tescan, Czech Republic). Ultraviolet–visible spectroscopy (UV–vis) (SP UV-26, SCO TECH, and Germany) was used to measure the transmission and absorption of samples in the visible-ultraviolet-near-infrared range to confirm the self-polymerization of dopamine. Fourier transform infrared spectrometer (FTIR) (PerkinElmer, Frontier, USA) was used to confirm the bonding between PDA-NPs and HA. In addition, dynamic light scattering (DLS) (Zetasizer Nano, Malvern, England) was used to measure the hydrodynamic size of particles. The inductively coupled plasma spectrometry (ICP-OES) (Vista- MPX, Varian- USA) was used to determine the concentration of absorbed and removed ions.

### Preparation of PDA/HA-NPs

62.105 mg of DH and 248.42 mg HA were dissolved in 10 ml of Tris buffer (100 mM, pH 8.5) and the mixture was stirred at 500 rpm for 8 h. The NPs solution containing the targeting agent was centrifuged at 11,000 *g* for 15 min. The supernatant was separated through a 0.22 µm syringe filter. The product was dialyzed with a dialysis bag (cutoff: 12,000–14,000 Da) against deionized water (DI) for 3 days and freeze-dried. PDA-NPs without HA were used as the control.

### Gd^3+^ loading on PDA/HA-NPs

Various weight ratios of Gd^3+^ per mg PDA/HA-NPs were prepared (0.4:1, 0.8:1, 1.2:1, 2:1, and 2.2:1). Briefly, a solution of PDA/HA-NPs was prepared in 25 ml deionized water with a concentration of 1 mg/ml. Concurrently, different amounts of Gd^3+^ (2, 4, 6, 10, and 11 mg) were dissolved in 5 ml of deionized water. After that, 5 ml Gd^3+^ solutions were mixed with 5 ml PDA/HA-NPs solution and dissolved at 500 rpm for 6 h. Then, the solutions containing PDA/HA-NPs coated with Gd^3+^ ions were filtered via a dialysis bag (cut off: 12,000–14,000 Da) against distilled water (100 mL) for 24 h to remove the unreacted ions. The number of unreacted ions was evaluated by ICP-OES analysis.

### Adsorption efficiency

For each weight ratio, the amount of adsorption (q_e_) and adsorption efficiency (% REM) of Gd^3+^ ions were calculated using Eqs. (1) and (2), respectively.

1 $$ q_{e} = \frac{{\left( {C_{i} - C_{e} } \right)}}{m} $$

2 $$ {\text{REM}} \left( \% \right) = \frac{{{\text{C}}_{{\text{i}}} - {\text{C}}_{{\text{e}}} }}{{{\text{C}}_{{\text{i}}} }} \times 100 $$

where, C_i_ is the initial concentration of Gd^3+^ ion in the solution (mg/L), C_e_ is the equilibrium concentration of Gd^3+^ ion in the solution (mg/L), and m is the adsorbent dosage of PDA/HA-NPs (mg/L).

### Equilibrium adsorption isotherm study

Different isotherm equations including Langmuir, Freundlich, and Dubinin-Radushkevich equations were used to study the Gd^3+^ ions equilibrium and adsorption model (Table 1).

### MRI and CT

At first, PDA/HA/Gd^3+^ NPs (prepared at a weight ratio of 2.2 mg Gd^3+^ ion per mg PDA/HA-NPs) were freeze-dried. For MRI and CT scans, different solutions containing 2, 4, 6, and 10 mg/ml Gd^3+^ ions were prepared using freeze-dried NPs. In addition, Barium sulfate and Dotarem at equal concentrations were used as commercial controls. For CT and MRI scans, the CT number (Hansfield number) and signal intensity (in MRI scan) of each concentration were respectively determined. In this study, relaxivity measurements were performed in a 1.5 T MRI scanner at room temperature. MRI sequences were run in a time to echo (TE) of 12 ms and repetition times (TRs) of 394, 500, and 585. The intensity values of the resulting images were measured through RadiAnt DICOM Viewer software. The T1 values were acquired by fitting a logarithmic curve to the intensity-TR diagrams. Finally, relaxivities (r_1_ in mM^-1^ s^-1^) were measured by the slope of the linear curve fitting to the 1/T1-CA concentration plot.

### Cytotoxicity assay

The cytotoxicity of PDA/HA/Gd^3+^ NPs (the weight ratio of 2.2:1) was investigated on HEK 293 cells via up-taking of thiazolyl blue tetrazolium bromide (MTT, Sigma) through the viable cells. The cells were plated into 96-well plates at a density of 1.0 × 10^4^ cells/100 µL/well. After incubation at 37 °C for 24 h, the medium was replaced with a medium containing different concentrations of PDA/HA/2.2Gd^3+^ NPs (0, 63.59, 158.98, 317.96, 635.93, and 1271.80 µM), and the plates were incubated for 72 h at 37 °C. The medium without NPs used a negative control. After that, 100 µL MTT solution (0.5 mg/mL) was added to each well and the cells were incubated for 3 h at 37 °C. Following solubilizing the precipitated formazan with 100 µL DMSO, the optical density was measured at a wavelength of 570 nm using a microplate spectrophotometer (Epoch, BioteK, USA). Additionally, to evaluate the potential toxicity of Dotarem as a control, concentrations from 0 to 1271.80 mM of Dotarem were investigated within 72 h.

### Hemolysis test

PDA/HA/Gd^3+^ NPs with different Gd^3+^ concentrations (2, 4, 6, and 10 mg/mL) were suspended in PBS solution and incubated for 30 min at 37 °C. Then, 200 µl healthy human blood diluted in PBS (at a ratio of 4:5) were added to 1 ml prepared NPs solutions and the mixtures were incubated for 1 h at 37 °C. The supernatants were separated with centrifugation at 2000 *g* for 5 min, and finally, the optical density of samples was measured at a wavelength of 550 nm by a spectrophotometer (BioTek, USA). PBS and distilled water were used as a negative and positive control, respectively. All experiments were repeated three times.

### Targeting study

To investigate the targeting of synthesized CA (PDA/HA/Gd^3+^), CD_44_^+^-overexpressing cells (MDA-MBA-231) were cultured in 6-well plates at a density of 3.5 × 10^5^ cells per well. After 16 h of incubation, the media were replaced with DMEM containing synthesized CA at three different concentrations (2, 4, and 10 mg/ml of Gd^3+^ ions). The cells were also treated with PDA/Gd^3+^ without HA (as negative control) at equal concentration. The cells are incubated for 4 h. After the incubation period was over, the cells were washed twice with PBS, then, the number of signals obtained from the Gd^3+^ ion was measured using MRI imaging. Relaxivity measurements were performed in a 1.5 T MRI clinical scanner at room temperature. MNP-Mn-PEG samples were prepared in deionized (DI) water. Then, MRI sequences were run for R1 measurements in a TE of 12 and TRs of 349, 500, and 585 ms. The signal intensity of resulting images was measured through RadiAnt DICOM Viewer software that calculates the mean MRI signals in the interest regions. The T1 values were obtained by fitting a logarithmic curve to the intensity-TR Plot. Finally, relaxivities (r_1_ in (mg/ml)^-1^ s^-1^) were acquired through the slopes of the linear curve fitting to the relaxation-concentration rate plot (R1 (1/T1)-C).

### Stability test for PDA/HA/Gd^3+^ CAs

The gadolinium releases of PDA/HA/Gd^3+^ NPs were investigated at a temperature of 37 °C. Synthetized NPs were dispersed at a concentration of 2, 4, 6, and 10 mg/ml at 5 ml of human serum, then dialyzed with a dialysis bag (cut off: 12,000–14,000 Da) against distilled water (100 mL) for 72 h by ICP-OES analysis^11^. The number of unreacted ions was measured with ICP-OES. Moreover, the stability of the PDA/HA/Gd^3+^ NPs was investigated by measuring DLS and the zeta potential after 3 days^43^.

### Statistical analysis

Plots for UV–visible, DLS, FTIR, adsorption studies, and plots related to MRI and CT scans were drawn with Origin Pro 2018. The plots for MTT and hemolysis assays were performed using Graph Pad Prism 8.0.2. The data for MTT and hemolysis assays were statistically analyzed using SPSS version 25. The processing of medical images was checked using RadiAnt DICOM Viewer 2020.2 software. The polydopamine NPs diameters were determined by ImageJ software (https://imagej.nih.gov/ij/index.html). The normality of data was evaluated with Shapiro–Wilk's normality test. The significant difference between groups was analyzed using Kruskal Wallis and post hoc tests. A *p*-value of less than 0.05 was considered a significant difference between the groups.

## Data Availability

All data generated or analyzed during this study are available from the corresponding author upon reasonable request.
